# Perspectives of adolescents, parents, and teachers on barriers and facilitators of physical activity among school-age adolescents: a qualitative analysis

**DOI:** 10.1186/s12199-019-0775-y

**Published:** 2019-04-09

**Authors:** El-Ammari Abdelghaffar, El Kazdouh Hicham, Bouftini Siham, El Fakir Samira, El Achhab Youness

**Affiliations:** 1 Laboratory of Epidemiology, Clinical Research and Community Health, Faculty of Medicine and Pharmacy, University Sidi Mohamed Ben Abdellah, Fez, Morocco; 2 Regional Centre for Careers Education and Training (CRMEF Fès-Meknes), Fez, Morocco

**Keywords:** Perspectives, Barriers, Facilitators, Adolescents, Physical activity, Morocco, Qualitative study

## Abstract

**Background:**

Physical activity levels are low among adolescents in Morocco; however, the influences on physical activity behavior of adolescents have not yet been explored in a qualitative study. Here, we explored potential social-ecological barriers and facilitators of physical activity in Moroccan adolescents with the goal of developing a successful intervention program aimed at improving their physical activity level.

**Methods:**

For this study, we conducted 17 focus group discussions (100 participants, composed of 56 adolescents, 26 parents, and 18 teachers from two middle schools in Taza city, Morocco). Discussions during focus groups were facilitated by a semi-structured interview guide. Guide questions were underpinned by the social-ecological model as a theoretical framework. Data analysis was carried out by two coders using thematic analysis.

**Results:**

We found that barriers and facilitators of physical activity in adolescents are organized into six themes that belong to different levels of the social-ecological model. Three themes belonged to the intrapersonal level (perceived motivating and limiting factors, physical activity awareness, and time constraints), two themes were classified into the interpersonal/cultural level (social support and gender and cultural norms), and one theme belonged to the environmental level (access to opportunities). Most of the themes were at the individual level, with each theme including both barriers and facilitators.

**Conclusions:**

Adolescent participation in physical activity can be facilitated or hampered by many factors. Results from the focus group discussions showed that these factors belonged to different levels of the social-ecological model, but most were at the individual level. Our findings have several implications. First, they may offer suggestions for a tailored intervention program aimed at improving adolescent physical activity. Second, they can improve quantitative research by enriching the battery of questions of physical activity instruments (e.g., a question related to physical disability). Third, the proposed thematic map can contribute to understanding interactions and causal pathways in the social-ecological model.

## Background

Adolescence is a period of biological and social transition, in which future patterns of adult health are established [1, 2]. Although most adolescents are perceived as healthy, important behavioral risk factors emerge during adolescence, and these behaviors can lead to disorders in adulthood [2, 3]. A decline in physical activity (PA) marks this period of life [4]. Worldwide, physical inactivity has become the fourth leading cause of global mortality. In 2008, more than 5.3 million of the 57 million deaths that occurred worldwide were attributable to physical inactivity [5]. Lack of PA is related to several noncommunicable diseases (including heart diseases, diabetes, and cancer), as well as other health problems [5, 6]. Maintaining adequate levels of PA among adolescents could substantially improve physical and mental health (as measured by, for example, cardiorespiratory and muscular fitness, bone health, cardiovascular metabolic health biomarkers, body composition, and psychosocial outcomes) [7–11]. Although burdens associated with physical inactivity are widely recognized, more than 80% of adolescents worldwide are physically inactive based on the World Health Organization-defined threshold [12]. According to their definition, to achieve health benefits, adolescents should accumulate at least 60 min of moderate to vigorous intensity PA every day [6]. Therefore, interventions to improve adolescent PA are needed, especially because PA habits adopted during adolescence can track to adulthood [13].

To develop an effective intervention, what is first needed is a better understanding of why adolescents (do not) engage in PA [14]. To reach this goal, ecological frameworks are particularly suitable because they consider the plurality and the complex interplay between the potential influences on PA [15]. Ecological models are a comprehensive approach that span factors affecting PA at multiple levels: the intrapersonal level (biological, psychological); the interpersonal/cultural, organizational, and physical environment level (built, natural); and the policy level (laws, rules, regulations, codes) [15].

Evidence from quantitative designs has shown that engagement of adolescents in PA is influenced by factors belonging to different levels of the ecological model, with determinants of adolescent PA mainly shown to be within the intrapersonal level. These determinants include age, sex, socioeconomic status, and psychological and biological factors [16–18]. In addition, several consistent influencing factors have been shown to be within the social and physical environments (including social support from family and friends and access to opportunities for PA) [16, 18–21]. PA research has been often dominated by quantitative approaches; however, qualitative studies have recently been rising. There are some complementary components between quantitative and qualitative approaches [22, 23]. But, qualitative research can offer additional understanding on key factors that facilitate “facilitators” and hamper “barriers” to adolescent participation in PA, as well as result in suggestions from adolescent and other participants on how to implement a successful intervention to improve PA levels in adolescents [24]. Findings from qualitative studies have shown that the key factors that influence adolescent PA are within the individual level (including enjoyment, perceived benefits, and motivation) [25–29] or within the interpersonal and cultural levels (e.g., social support) [25, 27–31] or even within the environmental level (e.g., accessibility and availability of PA opportunities) [25–29, 31].

Like many countries [12, 32], physical inactivity rates among Moroccan adolescents is alarming. In 2016, the Global School-based Student Health Survey reported that only 11.0% of Moroccan school-age adolescents are active [33]. Many factors are responsible for this situation; some of them are common with other counties, but others are specific to our Arab-Muslim culture society which seem particularly discouraging of PA [34]. The practice of PA is not a valued habit in Morocco for a variety of physical and cultural reasons [35]. There is a lack of parental support to PA, partly because parents favor educational success over exercise for their children [34, 35]. The practice of PA is known to manage weight, but in almost all countries in the Arab world, people tend to tolerate fatness, and there were no social sanctions against adiposity [36]. On the contrary, female plumpness is culturally preferred in some Moroccan ethnic group and even represents a symbol of beauty and prosperity [37]. Men are more active than women because of conservative social norms and cultural restrictions on outdoor activities and exercise for women [34, 35]. Although Morocco is undergoing an epidemiological transition [38], data on factors influencing PA among adolescents are lacking. Influencing factors of PA have never been explored among Moroccan adolescents using a qualitative design. Here, we conducted a qualitative study to identify potential social-ecological barriers and facilitators of adolescent PA.

## Methods

### Study design

To investigate social-ecological factors influencing adolescent PA, we collected qualitative data from participants in Taza city, a medium-sized city (207,984 inhabitants) in north-central Morocco [39], from February to July 2016. Specifically, semi-structured focus groups (FG) were asked about their perspectives and experiences related to factors that influence adolescent PA. Because triangulation of data sources guarantees the credibility of results from qualitative studies [24, 40], we included three types of participants: adolescents, parents, and teachers. The study was approved by the Faculty of Medicine and Pharmacy of Casablanca Research Ethics Committee and by the National Control Commission for the Protection of Personal Data (A-RS-193-2015) and authorized by The Provincial Directorate of Education Ministry in Taza. Informed consent was obtained from all parents and teachers. Adolescent consent forms were signed by their parents, but an additional verbal consent was also given by adolescent participants. Participant privacy was protected through anonymous and voluntary participation.

This qualitative study is part of a larger underway research project [41], which has a major goal of developing and implementing an intervention that supports a healthy lifestyle among adolescents. To reach this goal, potential factors influencing risk behaviors (e.g., PA and nutrition) were first identified through a mixed methods approach involving a quantitative study [42].

### Participants

Participants were recruited from two middle schools (disadvantaged and advantaged per socioeconomic level) that participated in the previous quantitative study [42]. From each school, 14- to 16-year-old students were invited to participate in FG discussions. This age group of students was selected because their curriculum includes topics related to health risk behaviors. Teachers were of the three school subjects that are concerned with health risk behaviors: “Physical Education,” “Life and Earth Sciences,” and “Islamic Education.”

Participant recruitment was significantly facilitated by directors and the general surveyor of schools. Adolescent, parent, and teacher participants first received an information letter specific for their group type. The letter provided a comprehensive explanation of study objectives and processes and contained an informed consent to sign. Second, after the participant informed consent was received, researchers, participants, and school officials selected an appropriate date and setting to conduct FGs. Unlike quantitative studies, the number of participants was not previously calculated, and sampling continued until data saturation was reached.

### Data collection

We chose the FG method because it encourages interactive discussions between participants [43]. Discussions were conducted by a moderator and an observer who are already trained to conduct FG discussions. The moderator managed the discussions with participants while the observer noted nonverbal cues (e.g., mimics, gestures, silence) and group interactions that seemed useful to understanding nonverbal participation. All FG discussions were conducted in the Arabic language, the mother tongue of the participants and interviewers.

The FG discussions followed a semi-structured interview that began with an introduction, followed by an opening question, and then open-ended questions that served to map further discussions to identify barriers and facilitators of adolescent PA (Table 1). Our FG guide, which was developed using the social-ecological framework as a theoretical framework [15], was pilot tested with a convenient sample of each type of participant and refined before definitive use. The total number of FGs was determined by the saturation principle.

**Table 1:** Focus group discussion steps

	Adolescents	Parents	Teachers
Step 1: Introduction	After the moderator and observer introduced themselves, the moderator stated the study objective and clarified anonymity, and some consigns to conduct discussion. Permission to record the interview was also obtained. Finally, PA was defined in detail.		
Step 2: Opening question	AQ1: Each of you describe your PA and then tell us how much time you should devote to PA each day to stay healthy?	PQ1: Please, can you tell us how much time your children can devote to PA each day to stay healthy?	TQ1: Please, what can you tell us about the PA level of adolescents today?
Step 3: Open-ended questions to identify barriers and facilitators of adolescent PA	AQ2: Please tell us the factors that help you to do PA? Give any factors, regardless of their nature. AQ3: Please tell us the factors that impede you to do PA? Give any factors that exist, regardless of their nature.	PQ2: Please tell us the factors that help your children engage in PA? Give any factors that seem relevant. PQ3: Please tell us the factors that prevent your children from engaging in PA? Here too, please tell us any factors, regardless of their nature.	TQ2: Please, what are the barriers to PA among adolescents? Give any factors that seem relevant. TQ3: How do you evaluate the role that the school plays in adolescent PA?

FGs were homogeneous regarding sex to overcome possible bias and to provide an opportunity for possible gender-specific factors to emerge [29]. In both schools, FG discussions were conducted in a suitable room where participants felt most comfortable and which allows a good quality audio recording. Participants also gave permission to record and use their anonymous quotes in research publications.

### Data analysis

Audio tapes from the discussion were fully transcribed and organized into separate datasets for adolescents, parents, and teachers. Relevant field notes such as nonverbal cues were also included in the transcribing process. All datasets and notes were analyzed by the coders using thematic analysis method of Braun and Clarke, which is a method for identifying, analyzing, and reporting themes within data [44]. Themes are patterns which capture something interesting about the data in relation to the research question [44]. Coders followed one-by-one the different steps proposed by Braun and Clarke: become familiar with the data, generate initial codes, search for themes, review themes, define themes, and write-up [44]. Coders started reading and re-reading the interview transcripts to become familiar with the data, took notes, and jot down early impressions. Then, open coding was used; that means we did not have preset codes but developed and modified the codes as we worked through the coding process. In addition, the coding process included consideration of our research question. So, this was an inductive-deductive thematic analysis. The next step was to search for themes; once the initial codes were generated, we started to categorize and combine them to form overarching themes. Themes were predominately at the semantic level, i.e., they are identified within the explicit or surface meanings of the data. After developing the initial themes, they were continually reviewed, created, and discarded using an iterative process. Thus, we constantly moved back and forth between the selected extracts from the data and the entire dataset to check if the themes make sense and account for all the coded extracts and the entire data set to assess the applicability of themes. Finally, each theme was defined, labeled, and analyzed. A thematic map was also established; it shows the organization of themes according to different levels of the social-ecological model and possible interactions between themes.

To ensure transparency and reliability, all transcripts were coded thoroughly by two researchers (the first and the second author of this paper) independently. Regular meetings with the research team were conducted to discuss and agree on any outstanding questions. The analysis team discussed their coding and interpretation of the transcripts in detail, and any coding differences were discussed and resolved to refine codes and identify key themes from the data. We translated into English the quotes used to illustrate themes and subthemes. No software was used in the data analysis.

## Results

A total of 100 people participated in 17 FGs. The estimated point of saturation was observed after 8 FGs of adolescents (*n* = 56, 50% boys), 5 FGs of parents (*n* = 26, 81% males), and 4 FGs of teachers (*n* = 18). Each FG discussion lasted between 50 and 80 min. Additional descriptive sample characteristics are presented in Table 2.

**Table 2:** Participant characteristics

Adolescents (*n* = 56)	
No. of participants per group	7
Sex (% boys)	50
Age range (years)	14 to 16
Grade level	3rd grade of middle school
Perceived family income	
Low (%)	5.36
Average (%)	94.94
High (%)	0.0
Education level of parents	
Fathers (% illiterate)	4.0
Mothers (% illiterate)	36.36
Parents (*n* = 26)	
No. of participants per focus group	5 to 6
Sex (% female)	19.23
Age range (years)	30 to 60
Education level (% illiterate)	0
Teachers (*n* = 18)	
No. of participants per focus group	4 to 5
Age range (years)	30 to 60

**Fig. 1:**
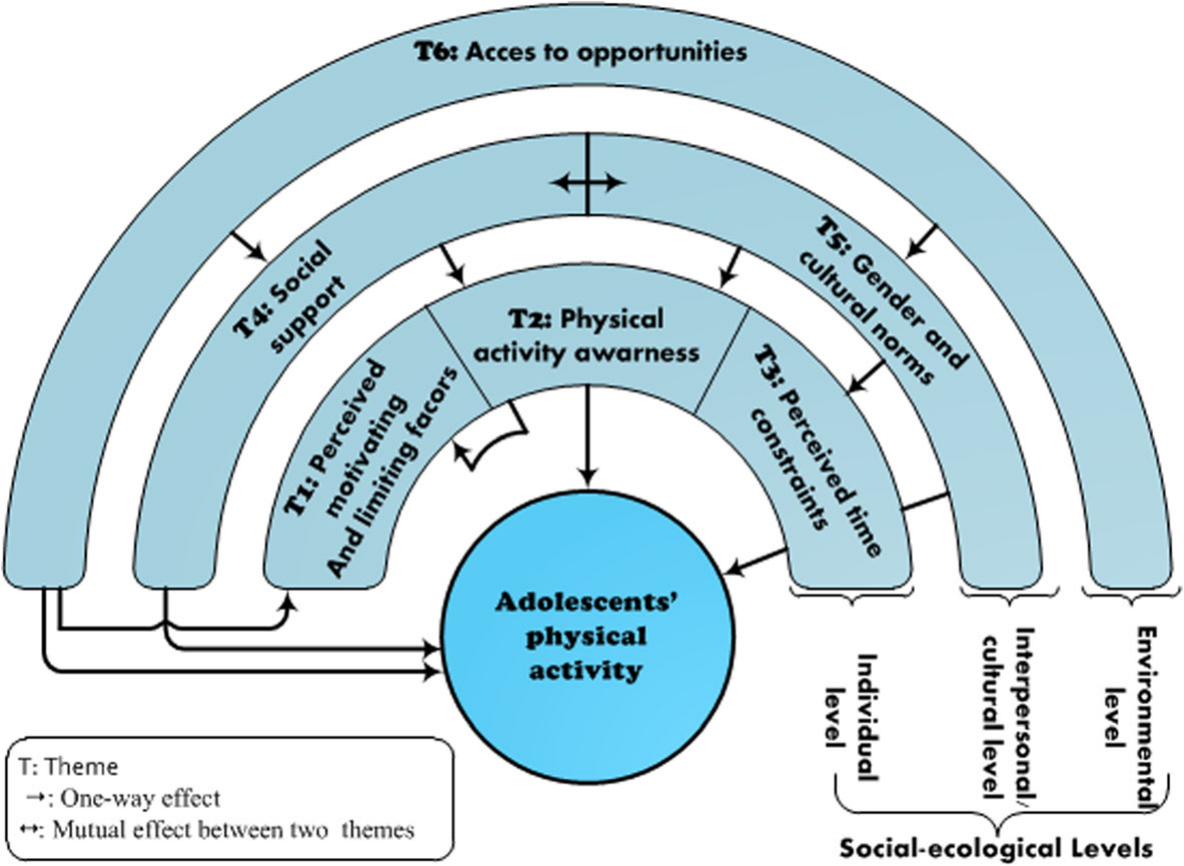
Thematic map showing identified themes and potential interactions between them

After data analysis, six main themes regarding the perceived barriers and facilitators of adolescent PA were identified: (1) perceived motivating and limiting factors, (2) PA awareness, (3) perceived time constraints, (4) social support, (5) cultural and gender norms, and (6) access to opportunities. Further descriptions of each theme and subtheme, as well as informative quotes in support of our results, are shown in Table 3. Themes 1, 2, and 3 were intrapersonal-level themes, themes 4 and 5 were interpersonal/cultural-level themes, and theme 6 was the single environmental-level theme. Figure 1 presents a thematic map that shows the organization of themes according to different levels of the social-ecological model and possible interactions between themes.

**Table 3:** Quotes from participants used to support themes and subthemes

Themes and Subthemes	Quotes		
	Adolescents	Parents	Teachers (T)
Theme 1: Perceived motivating and limiting factors			
Subtheme 1: Enjoyment and competition	- G11: “This sport pleases me a lot, there … I meet my friends and so I come out of the daily routine: from home to school and vice versa”	- M1: “He plays football with his friends, he wants to go out to entertain himself, ...”	- T1: “I see that during adolescence, boys prefer physical activities of their competitive nature”
Subtheme 2: Perceived physical and financial benefits	- B4: “I am exercising because sport is good for health and, … also to keep a good heart rate. Sometimes we play to earn some money”.	- F4: “they are obsessed with keeping a particular silhouette and body image especially among girls”	- T10: “Their only objective is to expose their bodies and fitness to show off in front of their friends”
Subtheme 3: Physical, psychological, and behavioral factors influencing PA	- G14: “It’s normal, ... it’s not my hobby, sport does not matter to me” B8: “..., there is laziness in first position …, there are some who have health problems like a physical disability, so they cannot practice PA”	- F16: “..., another reason is that there are some children who do not have certain skills to practice a given sport, ... for example, if he does not know how to play football, he will prefer not to play it to avoid embarrassment in front of his peers”	- T13: “Sometimes their physical ability pushes them to practice sport and sometimes their disability can prevent them”
Theme 2: PA awareness			
	- G7: “..., and I know it is beneficial for health” Moderator: “To be healthy, do you know how much of daily physical activity (duration) you must have?” - B15: “three hours per day” - B16: “Fifteen minutes per day”	- F2: “These adolescents who are active, you find them aware of the importance of PA for health”	- T9: “students who have less PA, the cause is that they are unaware of its importance” - T2: “Active children …, it is certain that they are aware of the importance of physical activity”
Theme 3: Perceived time constraints			
Subtheme 1: Loaded curriculum	- B27: “My free time, I dedicate it entirely to homework …, I do not do sport at all”	- F21: “The first thing is that there are some adolescents who say that sport is not essential compared to academic success, they do not see any importance for PA to achieve a good future”	- T2: “the matter is that the loaded curriculum does not give students an opportunity to do PA”
Subtheme 2: Time mismanagement and competing leisure sedentary activities	- G28: “…, The mismanagement of time because of many things ..., and ..., the misuse of the smartphone remains the main factor”	- F14: “Most of them are just sitting watching TV or surfing the internet” - F15: “They go out with friends to entertain themselves without practicing any PA”	
Theme 4: Social support			
Subtheme 1: Familial and friends influences	- G25: “There are numerous factors that motivate me to play sports ... there is encouragement of my parents especially that my father is an ex-sportsman, he has already participated in the marathon” - G15: “some parents do not enroll their children in sports clubs” - G4: “... and I do not have anyone with whom I can play”	- F6: “Encouragement from parents since childhood ..., for my children, I make sure that they do sports 3 to 4 times a week”	- T12: “Friends have a role in this …, for example when someone is enrolled in a sports club, he may encourage his friends to do the same”
Subtheme 2: Positive influences from others	- B6: “…, me, I hope someday that I will become like famous players of football like Ronaldo, I am a big fan of real club”	- F21: “There is also the influence of role models ..., for example, Messi or Ronaldo or others ...”	
Theme 5: Gender and cultural norms			
Subtheme 1: Gender differences	- G6: “I like to play sports, but my father does not allow me” - G21: “This because many people are not aware of gender equality …, people who believe in gender equality are a minority …” - G11: “My parents told me why you go there, that sport is for boys only, ...”	- F2: “Physical activity for girls is a problem …, they cannot play with boys, ... they will be intimidated by boys. There are no spaces reserved for girls ... I speak about spaces that are respectful, secure, no harassment, ...”	- T3: “In society, Customs and traditions do not accept that girls do sport” - T7: “Girls …, they are less active because they cannot play on the street like boys ..., girls are less interested in physical activity”
Subtheme 2: Cultural norms	- G21: “…, for example, girls should not do Gymnastic …, why? ... high jump too, why? ... normally there must be equality between girls and boys”	- F6: “Education based on religion and morality plays a role in this as it gives great importance to the preservation of body and health”	- T11: “The philosophy of sport that exists in developing countries, especially in the Arab countries is that people are encouraged to watch sports and not to practice it …, look for example at the great number of people who watch big matches but no one plays any kind of sports, they are all weak …, sport should be practiced and not watched”
Theme 6: Access to opportunities			
Subtheme 1: School physical Education classes	- G28: “Physical education classes are insufficient and sometimes they are scheduled between other school subjects”	- F16: “The problem is that even school environment is not encouraging and …, it does not allow adolescents to catch up the lack of PA they have outside school …, We teach physical activity but we do not encourage its practice”	- T12: “…, in PE classes, where there are no dressing rooms, girls are obliged to change clothes under the eyes of boys. So, girls are embarrassed as they can be seen by boys and therefore some of them apply for a certificate of exemption from PE classes”
Subtheme 2: Outside school opportunities	- G2: “I like basketball, but I cannot find where to play it except in PE classes sometimes”	- F12: “There is a lack of sports spaces that are equipped and secured against any harassment” - M1: “firstly, there is a lack of sports facilities. Something else ... the economic situation of some families does not allow them to enroll their children in suitable sports clubs”	- T13: “In our city, there is a lack of spaces to do sport …, if they exist, they are either expensive or they are not clean ..., for example there are gyms in cellars of buildings that have very poor ventilation …, it’s stinky” - T13: “…, But sports facilities do not exist and if they exist they are poorly supervised ..., so children are not encouraged to go there”

*G* girl, *B* boy, *F* father, *M* mother

### Theme 1: Perceived motivating and limiting factors

This theme refers to internal motivations and restrictions toward adolescent PA. It comprises three subthemes: enjoyment and competition, perceived health benefits, and perceived limiting factors.

#### Subtheme 1: Perceived enjoyment and competition

Expressions used by both male and female adolescents to support this subtheme included “*Sport pleases me a lot*,” “*I’m enjoying by doing sport*,” and “*I exercise to entertain myself*.” Having fun was a strong facilitator of adolescent PA. Some adolescents expressed a strong desire to exercise, with PA allowing them an outlet from daily routines and the ability to share enjoyment with friends. Parents and teachers also confirmed these statements. Competition was perceived as a facilitator for PA by some participants, which were almost exclusively boys.

#### Subtheme 2: Perceived health and financial benefits

Participants expressed that preserving physical and mental health in adolescence and in adulthood was a reason to engage in PA. Weight control, self-defense, physical appearance, and showing off muscles (among boys) to friends were also reasons for adolescents to engage in PA. Surprisingly, some adolescents practiced football for financial objective, contributing a sum of money that would be ultimately awarded to the winning team.

#### Subtheme 3: Physical, psychological, and behavioral factors influencing PA

Participants stated that these physical, psychological, and behavioral factors were overwhelmingly barriers to adolescent PA and included the presence of a physical disability, poor fitness, lack of skills in sports, lack of interest toward PA, laziness, timidity, and fear of accidents. Participants also mentioned that adolescents who smoke or use drugs are unmotivated to be active. Some parents and teachers had a different perspective and did not consider this subtheme as barriers to PA. Instead, they expressed that having some physical skills, having willingness, not having a disability, or being away from substance use were facilitators of PA.

### Theme 2: Physical activity awareness

This theme could be both a barrier and a facilitator of PA, depending on the level of adolescent awareness regarding PA. Awareness included having knowledge of adequate PA duration and intensity and the benefits on health (e.g., physical and psychological health, disease prevention). Participants stated that media, family, and school play a role in raising PA awareness, although some participants argued that awareness played only a modest role. Parents and teachers highlighted that young people were often disinterested toward advice from others.

### Theme 3: Time constraints

This theme comprised two subthemes, both being barriers to PA: (1) overloaded curriculum and (2) time mismanagement and competing leisure sedentary activities.

#### Subtheme 1: Overloaded curriculum

Many participants mentioned that overloaded curriculum and homework greatly reduce adolescent leisure time, thus reducing the participation of adolescents in PA. In addition, some adolescents favored educational achievements versus participation in PA.

#### Subtheme 2: Time mismanagement and competing leisure sedentary activities

Mismanagement of time and not devoting a place to PA in the daily schedule of adolescents represented a barrier to PA. Many participants stated that they waste substantial time in front of a screen (including television, smartphone, tablet, or laptop), performing household tasks (for girls), or spending time with friends.

### Theme 4: Social support

Active adolescents had support from family members, which was also confirmed by parents and teachers. Parental support included encouragement and incentive to exercise, enrolment in sport clubs especially at an early age, and parental modeling. In addition, a family atmosphere without problems or conflicts supported adolescent PA. Support from friends was also stated by participants as a facilitator of adolescent PA. In addition, some adolescents practiced PA just to meet and spend time with friends.

Many participants highlighted that lack of support from parents, family, or friends has a negative influence on adolescent participation in PA. Lacking PA awareness in the family, not having friends, having friends who are inactive, or having friends with other interests or negative interests (e.g., substance use) keep adolescents away from practicing PA.

Some participants expressed that role models could also include successful national sports figures who may inspire adolescents to engage in sports.

### Theme 5: Gender and cultural norms

#### Subtheme 1: Gender differences

Girls, parents, and teachers explicitly reported that girls are particularly underprivileged in PA engagement compared with boys. Indeed, some girls stated that their parents prevented them from practicing PA. In addition, boys tended to take over neighborhood spaces with sports activities, especially football. Some girls expressed that it was shameful for them to participate in outside PA. Girls expressed spending almost all their leisure time at home, doing homework or helping their mothers in housework chores.

Girls and parents stated that there is a lack of appropriate spaces specific for girls that are secure, safe, and well supervised. There is a fear of being intimidated, harassed, or even assaulted. A final opinion that was emphasized by some teachers is that girls are less interested in PA than boys.

#### Subtheme 2: Cultural norms

According to the participants, some sports are perceived to be valid only for boys. They also argued that gender equality was not yet implemented in the society. Some teachers referred to customs and traditions as barriers to PA participation among girls. In addition, some parents and teachers stated that even if religion encourages participation in PA, a culture of PA is not common in the society. FG discussions also showed that young people watch sports incessantly through media outlets without practicing any kind of it.

### Theme 6: Access to opportunities

#### Subtheme 1: School physical education classes

Physical education (PE) classes are sometimes the only opportunity to practice PA. According to some participants, PE classes are insufficient (2 h per week), in fact, not meeting the PA threshold recommended for adolescents. In addition, PE classes face several difficulties. Participants state that PE classes are forbidden during bad weather; some adolescents stated that playgrounds were insufficient and unpractical. In addition, participants stated that PE classes are scheduled between other school subjects. PE classes at school also necessitated dressing rooms, which are often unavailable. Participants reported that girls were unwilling to participate in PE classes when there are no dressing rooms. Because girls are embarrassed as they can be seen by boys, they often apply for exemption from PE classes.

#### Subtheme 2: Outside school opportunities

Most participants indicated that there is a significant lack of sports facilities in their community. The rare spaces that exist are distant, expensive, or inconvenient. Participants also stated that these spaces are faced with many difficulties, including lack of security, lack of supervision, lack of equipment, harassment (particularly for girls), and poor ventilation for closed spaces. Such problems make these spaces impracticable. Participants also indicated that in the absence of suitable spaces, some adolescents play football in the street. Both girls and parents expressed the lack of spaces for girls. According to participants, addressing the lack of sports facilities is not a priority for local authorities.

Availability and accessibility of spaces (e.g., not distant, unfilled, less expensive, having family income to participate) have been considered significant facilitators of adolescent PA. Some participants stated that there are sufficient places to play football but no space for other types of sports (e.g., basketball, tennis, cycling, and swimming).

## Discussion

By using a qualitative design, our goal was to explore potential barriers and facilitators of adolescent PA. We identified that influencing factors were organized into six themes classified into different levels of the social-ecological model, with three themes belonging to intrapersonal level (perceived motivating and limiting factors, PA awareness, and perceived time constraints), two themes classified into interpersonal/cultural level (social support, and gender and cultural norms), and one theme corresponded to the environmental level (access to opportunities). Such themes include both barriers and facilitators of PA, which have been reported previously [28, 31]. Interactions between the themes, as shown in the thematic map (Fig. 1), can provide valuable insights to identify possible mechanisms to improve PA. Because reports on interactions and causal pathways within the social-ecological model regarding PA are scarce [45], these findings could be useful in the development of a tailored intervention aimed at improving adolescent PA.

### Intrapersonal factors

As reported previously [25–29, 31], positive expectations in PA, such as having fun, spending time with friends, and breaks to daily routine, were important facilitators of adolescent PA. Similarly, all participants considered that health benefits, weight management, and preserving a particular physical appearance were important reasons to engage in PA. These factors were also identified in previous studies [25–28, 31]. In literature, competition is often considered as a barrier to adolescents’ PA especially among girls and those lacking skills or physical conditions [25, 26, 28, 46]. In our study, competition was perceived as a facilitator for PA by some participants, which were almost exclusively boys. A likely explanation for this result may be that these boys have good skills or physical conditions, which encourage them to enjoy physical activities of a competitive nature. Self-defense, against an aggressor for example, was a reason for some adolescents (especially girls) to engage in self-defense sports, expressing feeling unsafe within their neighborhoods. In line with a widely known custom among boys but unusual in the literature, many boys stated that they play football to win money. Hence, organizing sports competitions with prizes to the winning teams could encourage PA in boys.

Similar to previous studies, adolescents with physical disability [47] and/or with poor physical condition and skills [25, 27, 28, 31, 46] engage in less PA than their normal peers. In a systematic review, Carlon et al. found that youth with cerebral palsy participated in 13 to 53% less PA than their peers and were more sedentary [47]. Long-term negative health consequences of inactivity among disabled youth are therefore more likely, which can in turn reduce their life expectancy [47]. Therefore, intervention programs that seek to increase the PA level among this category of population are needed. Interventions can target some modifiable physical, psychological, and environmental correlates identified in previous studies [48]. However, it was concluded in two recent systematic reviews [49, 50] that the effectiveness of this kind of interventions was not always sure, and future studies are required before giving recommendations for such interventions. In the literature, physical disability was rarely reported as a barrier, especially in relevant systematic reviews of quantitative studies [16–18, 20]; this may be because questions related to disabilities were not included in questionnaires measuring PA. Thus, considerations of physical disability in the development of such tools could prevent possible biases in the estimation of PA level.

Similar to a number of previous studies [27–29, 46], our results showed that participation in PA decreases negative psychological characteristics such as low desire and importance, poor enthusiasm toward PA, and laziness, timidity, and fear of accidents. Thus, improvement of these mediators can positively affect adolescent PA level.

Smoking was perceived as a barrier to PA by participants of this study. This behavioral factor was not identified as a barrier in qualitative studies, although results from quantitative studies are inconclusive [16]. Furthermore, in a longitudinal cohort study, Audrain-McGovern et al. concluded that adolescent smoking uptake depended on the kind of PA; some types of PA are negatively associated with smoking uptake, whereas others are positively associated [51]. In studies supporting a negative association between PA and smoking, the underlying mechanisms are not yet understood. Thus, Audrain-McGovern et al. found that PA reward as one mechanism by which PA can reduce the likelihood of adolescent smoking uptake [52]. In the same sense, Verkooijen et al. conducted another study aimed at gaining a better understanding of this association and found that some motives like friendships and competition appeared to moderate the association between PA and smoking rather than others (e.g., weight management and self-esteem). This study recommends focusing on first motives and avoid the last ones when designing a smoking prevention program [53]. Finally, the relationship between PA and smoking is complex, further research is warranted to identify other mechanisms underlying this relationship to provide evidence-based smoking prevention targets [53].

Adolescents and parents were generally aware of the health benefits of PA. In line with previous studies [29, 54], this was considered as a facilitator of PA practice. Regarding adolescent PA guidelines, most adolescents and parents did not know that adolescents must accumulate at least 60 min of daily moderate to vigorous intensity PA to stay healthy. Being unaware of this threshold may lead to an overestimation of PA level among adolescents, believing themselves to be more active than they really are [55]. In the same way, parents who overestimate the PA level of their children may see no need to encourage them to increase their PA [55]. Improving awareness related to PA health benefits and recommendations among adolescents and their parents may encourage behavior changes in adolescents [55]. Furthermore, involving media, school, and family may be useful in raising PA awareness among adolescents because participants considered them to be the main awareness-raising sources of PA.

Our study results agree with previous findings that lack of time was a strong barrier to adolescent PA [25, 28, 29]. Participants linked the lack of time to school demands (e.g., overloaded curriculum, private lessons, homework, and giving higher priority to academic success), screen device use, time spent with friends, time mismanagement, and household tasks (among girls). Improving the adolescent’s time management skills should be targeted when setting up an intervention program. Data from the latest Global School-based Health Survey indicated that 32.9% of Moroccan adolescents spent 3 or more hours per day on sedentary activities, not including time in school or doing homework [33]. It is proven that sedentary behavior and PA are two different constructs and that they do not directly displace one another [56]. Therefore, efforts to encourage decreases in sedentary behavior time and increases in PA must go together [56].

### Interpersonal/cultural factors

Social support has been consistently associated with adolescent PA [21, 57]. This support is provided by different sources (parents, family, friends, and others) and in different ways, either tangibly (e.g., doing activity with, watching/supervision, transportation, payment of fees) or intangibly (e.g., encouragement and praise, discussing benefits, role models) [20, 30, 57]. Similar to previous studies [28, 29, 58], our findings showed that tangible and intangible social support provided by parents, family, friends, and sometimes by national sports leaders was a salient facilitator of adolescent PA. However, an unsupportive social environment was a barrier to PA. Results also indicated that not having friends or having friends who are physically inactive or with other interests and negative interests (e.g., drug use) may keep adolescents away from PA. Therefore, social support should be targeted in intervention programs aimed at increasing PA levels among adolescents.

Girls are less active than boys, but the reasons behind this are not well understood [59, 60]. These gender differences were explained previously by non-modifiable variables, including a girl’s biology [61], and by some modifiable variables related to a girl’s psychology [60, 62, 63], social support [59, 60, 62], or cultural and physical environmental factors [60, 62]. Our use of single-gender FGs and involvement of parents and teachers proved useful in identifying barriers that led to inactivity in girls. Findings showed a variety of barriers mainly of a sociocultural aspect. Similar to most Arab countries [34, 64], girls received less social support compared with boys, as many families do not allow their girls to practice PA outdoors because of conservative cultural and religious norms. To preserve public modesty, many parents do not permit their girls to practice PA with sports dress, but with traditional dress, which is not suitable for PA [37, 64]. Some traditional long dress covering the whole body may hide women fatness and consequently reduce their motivation to do PA [36, 37, 64]. In addition, cultural expectations concerning women’s roles were considered as barriers to PA. Thus, like many countries in the world, Moroccan women were expected to care for the family and household and their exercise needs were afforded low priority [34, 35]. Participants reported that some sports were socially perceived to be valid only for boys, and there is also a lack of appropriate spaces specific for girls that are secure, safe, and well supervised. In a review on Arab adolescents, Obermeyer and associates indicated that some features of social context such as unequal gender norms can affect adolescent health [38]. Similarly, the recent Arab Human Development Report indicated that gender equality and women’s empowerment are more restricted in Arab regions than in other regions [65]. In brief, the identification of modifiable factors that explained gender differences in adolescent participation in PA is important since they can be targeted in interventions [62]. The mediators found in this study can be targeted to minimize the effects of gender and thus improve PA levels in girls. Nevertheless, objective measurements are required to ensure that they are relevant mediators because previous studies concluded that among a multitude of mediators examined, only some could explain the association between gender and PA [59, 62].

### Environmental factors

Various theoretical frameworks have been used to understand factors influencing PA. In the previous decades, the use of ecological models revealed numerous environmental factors that may affect adolescent PA levels. Identification of such factors is useful because young people are especially susceptible to environmental influences given that they have relatively little autonomy over their own behaviors [59]. Furthermore, many studies showed that interventions that affect environmental factors, along with other personal and interpersonal factors, are more likely to be effective [66]. This study showed that PE classes in school and recreational facilities outside school are two opportunities available for adolescents to practice PA.

Similar to previous studies [26, 27], our results highlighted that PE classes contributed to improving the overall PA level among adolescents. However, PE classes face several obstacles related mainly to infrastructure, equipment, schedules, and insufficient hourly volume. In such context, students, especially girls, apply for a certificate of exemption from PE classes. This negative impact of PE classes on PA levels in female adolescents was previously reported by other studies [25, 27]. Consequently, to improve adolescent PA, PE classes must be reinforced because it touches a high number of adolescents regardless of their social differences.

Outside school, participants stated that there is a lack of recreational facilities for many sports (e.g., jogging, cycling, basketball, tennis, swimming). The rare existing spaces, for football mainly, were often distant, insecure, unsupervised, not equipped, or expensive, often making these spaces impracticable. These issues were more pronounced among girls. According to the participants, a lack of opportunities outside the school was because addressing these issues was not a priority for city policymakers.

Participant recruitment in this study was limited to two middle schools in an urban area. Thus, it is not possible to generalize findings to a larger population. However, our goal was to generate a deep exploration of influencing factors of adolescent PA and not to generalize results. However, our high number of participants, as well as triangulation of participants, allowed us to obtain a more complete overview. Because of the qualitative nature of this research, results are not equal to objectively measured data. Thus, it was not possible to define which influencing factor was most significant for adolescent participation in PA. Furthermore, we did not give the percentages of participants reporting a theme or subtheme. As reported by Braun and Clarke [22], the issue raised most frequently or by a larger number of participants is not necessarily the most pertinent to answer the research question. Finally, the interactions between themes, as shown in the thematic map (Fig. 1), require an objective evaluation to prove their strength.

## Conclusions

This qualitative study is the first in Morocco to explore the identification of barriers and facilitators of adolescent PA. Overall, our findings show that barriers and facilitators are organized into six themes (perceived motivating and limiting factors, PA awareness and perceived time constraints, social support, gender and cultural norms, and access to opportunities). These themes included both barriers and facilitators of PA and belonged to different levels of the social-ecological model. Our findings have several implications. First, they could be used to develop tailored intervention programs aimed at improving adolescent PA. Second, they can improve quantitative research by enriching the battery of questions of PA instruments (e.g., a question related to physical disability). Third, the proposed thematic map (Fig. 1) can contribute to understanding interactions and causal pathways in the social-ecological model. However, these interactions require an objective evaluation to prove their strength.

## Abbreviations

FG: Focus group
PA: Physical activity
PE: Physical education
